# The dyadic regulation approach of coping and illness representations in female cancer patients and their partners

**DOI:** 10.3389/fpsyg.2023.1194900

**Published:** 2023-09-06

**Authors:** Zoe Giannousi, Christoforos Thomadakis, Evangelos C. Karademas, Antonia Paschali

**Affiliations:** ^1^Department of Basic and Clinical Sciences, University of Nicosia Medical School, Nicosia, Cyprus; ^2^Department of Psychology, School of Social Sciences, University of Crete, Rethymno, Crete, Greece; ^3^Faculty of Nursing, School of Health Sciences, National and Kapodistrian University of Athens, Athens, Greece

**Keywords:** dyadic regulation, dyadic coping, female cancer patients, psychological adaptation to cancer, psychooncology

## Abstract

**Purpose:**

Adjustment to any illness is a ‘dyadic' process whereby patients and their partners mutually determine each other's perceptions, behaviours, and well-being. The present study explored the association between dyadic coping strategies and illness representations in newly diagnosed female cancer patients and their partners.

**Methods:**

The sample consisted of 92 female cancer patient-partner pairs from 3 oncology hospitals in Greece and Cyprus. The Actor Partner Interdependence Model was applied to test for dyadic regulation effects.

**Results:**

The findings revealed that patients' evaluations of dyadic coping were related to their own illness representations and, in some cases, to partners' illness representations of control. However, partner evaluations of dyadic coping were not associated with either patients' or their own illness representations. Relationship satisfaction did not moderate the relationship between dyadic coping and illness representations.

**Implications:**

The study suggests that patients' perceptions of support provided by themselves and their partners play a significant role in shaping their illness representations. Future research could delve into the underlying reasons for the observed differences in the impact of dyadic coping on illness representations between patients and partners, considering factors such as gender roles and specific gender-related issues.

## Introduction

For couples, psychological adjustment to cancer is well-documented and broadly studied. The great changes, imposed by the cancer experience on both individuals and their families, cause stress, and the psychological adaptation is challenging for the whole family (Andersen and Simonelli, 2007; Applebaum and Breitbart, 2013). The coping strategies both patients and partners adopt in response to this stressful condition (i.e., a cancer trajectory) are mutually influenced and regularly re-appraised and adjusted (Karademas, 2021).

Partners are also affected by these serious diagnoses, and their adaptation to the new stressful situation is linked to the patients' psychological wellbeing. A meta-analysis by Hagedoorn et al. (2008) found a significant association between cancer patients' and their partners' distress. Moreover, Jacobs et al. (2017) suggested that the psychological symptoms of patients newly diagnosed with incurable cancer and their primary caregivers were positively correlated. Surprisingly, higher levels of anxiety were found in partners in comparison to patients.

Moreover, there is a growing body of evidence that suggests that the experience of an illness is not an individual or isolated process (Weinman et al., 2003). Patients' families and, especially, their partners or spouses are directly involved in disease management, patient support, decision-making, etc. They are also affected by the experience of illness and its multifaceted impact (Weinman et al., 2003). In a parallel and dynamic process, partners/spouses develop their own ways of perceiving and managing the situation which, in turn, interacts with patients' representations (Weinman et al., 2003). In other words, adjustment to illness is a 'dual' process in which patients and their 'significant others' mutually determine each other's reactions, health, and quality of life (Badr et al., 2010).

Not only partners are adjusting to the cancer trajectory by understanding and managing the situation themselves in a parallel way with patients, but also their representations seem to be interconnected and mutually determined by the patients' representations and vice versa (Weinman et al., 2003). As cancer has been widely associated with negative psychological outcomes, the need to better understand the various factors involved in how individuals psychologically adjust to the new, often devastating, life changes caused by the diagnosis and treatment of cancer is of the utmost importance (Richardson et al., 2017).

Two key factors have been suggested as playing a vital role in patients' adaptation to chronic illness, both in short and long term. The first important factor refers to the way patients perceive and understand their health status, i.e., the illness representations they form (Leventhal et al., 1980). The common-sense model explains the self-regulation process regarding how individuals comprehend illness before the diagnosis and during the illness trajectory, as well as their relationship to psychological and illness outcomes (Leventhal et al., 1980). Representations of illness are divided into components, such as “identity” (i.e., the symptoms), “consequences” (i.e., how severe it is), “timeline” (i.e., how long it will last), “contro” (i.e., what can be done to manage the disease on a personal or treatment level), and “causes” (i.e., what can have caused the disease). Representations of control are suggested to be strongly related to illness outcomes in cancer populations (Richardson et al., 2017). In a recent meta-analysis by Hagger and Orbell (2017), less controllable and more emotionally burdening representations of cancer were found to be more consistently related to lower levels of functional outcomes. Moreover, results from another study in cancer patients showed that most of them tended to have the sense that the illness would last longer and that sense was also related to poorer outcomes (Hopman and Rijken, 2015). Accordingly, partners form their own illness representations about the condition (Weinman et al., 2003). Several studies have suggested a significant relationship between partners' and patients' representations. Partner representations are likely to act for the patients as sources of additional information about their condition and thus may also affect the development of patient representations (Karademas et al., 2018). Several studies have also suggested that partner representations affect the psychological wellbeing and adaptation process of both members of the relationship (Dempster et al., 2011; Wu et al., 2013; Giannousi et al., 2016; Karademas et al., 2018).

The second important key factor in patients' adaptation process is coping. According to Lazarus and Folkman (1984), individuals adopt coping behaviors in order to manage the consequences arising from a stressful situation (e.g., a disease) at both an emotional and a practical level. Coping in cancer patients has been widely studied, and there is a growing body of evidence regarding the importance of coping behaviors in adaptation to cancer (e.g., Brandão et al., 2017; Dunne et al., 2017; Morris et al., 2018; Siwik et al., 2020). However, besides the patient's individual efforts to cope with illness, dyadic coping (DC) is also a crucial determinant of adaptation. According to Bodenmann et al. (2016), DC refers to a person's attempt to help relieve their partner's stress as well as the common efforts of both parties to cope with external stressors (such as illness). Several studies have examined the relationship between DC and the wellbeing of both patient and partner. For example, it has been found that overprotectiveness in a partner is related to worse psychological outcomes in cancer patients, whereas a partner actively engaging in conversations with the patient about their experiences has been related to better psychological outcomes for the patient (Hagedoorn et al., 2000; Kuijer et al., 2000). Moreover, more “positive” DC, such as problem solving and emotion regulation, as well as common coping (i.e., the involvement of both partners in the effort to cope with illness), have been positively associated with patients' psychological health (Badr et al., 2010; Acquati and Kayser, 2019).

The general aim of the present study was to examine, using a dyadic approach, the relationship of DC strategies with illness representations in a sample of newly diagnosed female cancer patients and their partners. According to the Common Sense Model which is constantly evolving (e.g., Cameron et al., 2005; Orbell et al., 2006; Karademas et al., 2011; Moss-Morris, 2013; Leventhal et al., 2016; Hagger et al., 2017; Benyamini and Karademas, 2019; Durazo and Cameron, 2019; Orbell and Phillips, 2019), patients form their representations about illness from all their previous experiences in life, as well as from the information they have received up to the diagnosis, available coping resources, etc. In other words, illness representations are formatted through the holistic context of all the patient's personal experiences rather than only through the experience of the illness *per se* (Leventhal et al., 1997, 2016).

This complex information activates multiple representations and coping reactions. In turn, these representations and the coping reactions are constantly re-appraised and evaluated by the individuals in a dynamic and iterative procedure until the best fit between representations, coping, and outcomes is achieved. In this sense, Hagger et al. (2017) supported the idea that coping plays a significant role as a moderator of the relationship between illness perceptions and outcomes. Therefore, it can be argued that coping strategies are part of the general context of factors that help individuals formulate their representations of illness.

In this regard, DC may also affect illness representations. DC involves both partners working together to cope with stressors like a cancer diagnosis. Through supportive behaviors and problem-solving discussions, this collaborative approach enhances individuals' sense of control over the illness. By believing they have the internal and external resources to manage the challenges, both the patient and the partner experience a greater sense of control. This, in turn, may not only improve psychological wellbeing and adjustment to cancer but also impact the ways that both partners understand the whole experience. That is, their representations about illness (e.g., as more controllable, less fearful, or shorter) or treatment (e.g., as more effective).

In this study, the aim was to investigate the connection between positive DC strategies (i.e., the support and help provided by self to partner, from partner to self, and common efforts to cope with the situation) and certain key illness representations among both patients and their partners in a dyadic regulation approach. Specifically, the focus was on examining how the DC strategies adopted by the couple were associated with the individuals' perceptions of personal control, treatment control, and timeline related to the illness. These components were chosen because they have been found to strongly impact health outcomes in cancer patients, as previously documented in studies by Hopman and Rijken (2015) and Richardson et al. (2017). The second specific aim was to examine the potentially moderating role of relationship satisfaction in the link between DC and illness representations. Relationship satisfaction is an important factor as far as DC and adaptation to illness are concerned. For example, a meta-analysis found that DC strongly predicts relationship satisfaction (Falconier et al., 2015). Another recent study in breast cancer patients and their partners showed that DC and marital satisfaction predicted posttraumatic growth while also moderating the impact of DC on posttraumatic growth for both partners (Suo et al., 2021).

Given that available coping and support resources are part of how a stressful condition is evaluated (Lazarus and Folkman, 1984), and in accordance with previous research on the relationship between representations of control and DC (Karademas et al., 2018), our first hypothesis was that a sense of more support received by the other member of the couple would be related to a more positive representation of illness. More specifically, higher levels of perceived (personal and treatment) control over the illness for both members of the couple, but also a lower timeline (i.e., the sense that the illness will last for a shorter period of time). Likewise, to the extent that the (dyadic) support provided by *self* to the other member of the couple also reflects the broader sense of support available in the couple, we expected its relation to the illness representations to have the same direction as the one described above.

Moreover, given the importance of relationship satisfaction in adaptation to illness and DC (e.g., Falconier et al., 2015; Suo et al., 2021), our second hypothesis was that relationship satisfaction moderates the relation of DC to illness representations for both members of the couple. We would expect that when both partners report higher levels of relationship satisfaction, the positive associations between supportive and common DC and positive illness representations (i.e., representations of control) would be stronger than when they report lower levels of relationship satisfaction. On the other hand, we expected the negative association between DC and negative illness representations (i.e., timeline) to be weaker at higher levels of relationship satisfaction.

## Methods

### Participants and procedure

The study was conducted at the Departments of Medical Oncology in two public hospitals in Greece and one in Cyprus. Female patients who were diagnosed with cancer < 3 months prior were invited to participate with their partners. Inclusion criteria for the patients included being over 18 years of age, having a first-time cancer diagnosis, being able to understand the study protocol, and providing informed consent. The hospital's medical files were used to identify eligible participants. Inclusion criteria for the partners were being over 18 years of age, not suffering from any severe illness at the time, and being able to provide informed consent. Additionally, participants had to have been in the relationship for a period of at least 1 year before the diagnosis. A research assistant approached patients who met the inclusion criteria while they were waiting for scheduled appointments at the clinic and invited them, together with their partners, to participate in the study. The patients and their partners who agreed to participate were asked to respond to the study questionnaires separately. The study was approved by the University of Crete Ethics Committee (No. 224/19-12-2019).

A total of 126 patients were identified as eligible for participation. In all, 34 patients or their partners refused participation because they were not interested, felt unable to participate, or provided incomplete data. The final sample consisted of 92 couples. All patients were married or living with their male partners for at least 2 years. Patients' mean age was 49.37 years (SD = 10.52 years). Details regarding the demographic characteristics of the participants and the disease characteristics of the patients are presented in Tables 1–4.

**Table 1:** Cancer diagnosis.

**Brest cancer**	**81.5%**
Gastrointestinal cancer	7.6%
Gynaecologic cancer	4.3%
Other types	6.6%

**Table 2:** Stage of cancer at diagnosis.

**Non metastatic cancer**	**Metastatic cancer**
83 (90.2%)	9 (9.8%)

**Table 3:** Treatment.

**Chemotherapy alone**	**31.1%**
Combination of treatments	53.3%
Radiotherapy or other therapies alone	15.5%

**Table 4:** Education.

	**Patients**	**Partners**
Nine year mandatory education	12%	17.9%
High school	40.3%	46.2%
Higher education Degree	47.7%	35.9%

### Measures

#### Illness representations

Illness representations were assessed with the Revised Illness Perception Questionnaire (IPQ-R; Moss-Morris et al., 2002). Three dimensions of patients' illness representations were assessed: personal control (6 items; e.g., The course of my illness depends on me; Cronbach *a* = 0.68), treatment control (5 items; e.g., My treatment can control my illness; Cronbach *a* = 0.79), and timeline (5 items; e.g., My illness will last a short time; Cronbach *a* = 0.80). A slightly re-worded version was used to assess spouses' representations regarding patients' illnesses (e.g., My actions have no effect on the outcome of my partner's illness. Cronbach *a*s for the three scales were 0.83, 0.75, and 0.82, respectively). To answer the questionnaire, respondents used a 5-point Likert type scale ranging from 1 (strongly disagree) to 5 (strongly agree).

#### Dyadic coping

DC was assessed with the Dyadic Coping Inventory (DCI; Bodenmann, 2008), as adapted in Greek (Roussi and Karademas, 2016). Three DCI subscales were used in the study. (a) The overall problem- and emotion-focused supportive DC provided by the other member of the couple as reported by the patients and their partners (4 items, e.g., My partner expresses that he/she is on my side; Cronbach's α = 0.85 and 0.79, for patients and partners, respectively). (b) The overall problem- and emotion-focused supportive actions provided by self to the other member of the couple as reported by the patients and partners (4 items, e.g., My partner expresses that he is on my side; Cronbach's α = 0.71 and 0.79, for patients and partners, respectively). (c) The overall problem and emotion focused common coping (i.e., what both partners do together to cope with an adversity; 5 items, e.g., We help one another to put the problem in perspective and see it in a new light; Cronbach's α = 0.87 and 0.90, for patients and partners, respectively). Participants were asked to respond with regard to the stressful conditions in general by using a 5-point Likert scale ranging from 1 (very rarely) to 5 (very often).

#### Relationship satisfaction

The Relationship Assessment Scale (Hendrick et al., 1998) was used to assess relationship satisfaction. The Relationship Assessment Scale is a 7-item measure (e.g., In general, how satisfied are you with your relationship?) that assesses a couple's relationship satisfaction using a 5-point Likert scale ranging from 1 to 5, with higher scores indicating more satisfaction (Cronbach's α was 0.90 for men and 0.88 for women).

#### Analyses

A MANOVA was performed to examine the potential impact of a series of sociodemographic and illness-related factors on the dependent variables. Specifically, the potential impact of the type of diagnosis (breast vs. other types of diagnosis), cancer stage, treatment type (single vs. combination of treatments), and patient and partner education level were examined. Pearson (*r*) correlations were used to examine the relationship between patient and partner age and duration of the relationship for all variables.

The Actor-Partner Interdependence Model (Kenny, 1996; Kenny et al., 2006; APIM) was used to examine the dyadic (actor and partner) effects of DC on illness representations. According to APIM, the relation of a person's independent variable to their own dependent variable is referred to as the *actor-effect*, whereas the relation to a partner's dependent variable is referred to as the *partner-effect*. A free user-friendly web application, the APIM_SEM (Stas et al., 2018; https://apimsem.ugent.be/shiny/apim_sem/), was used to perform the APIM analyses. APIM_SEM automatically performs the appropriate statistical analyses (i.e., structural equation modeling with maximum likelihood estimation using the programme; Rosseel, 2012). In addition, another free web application, the APIMoM (Kenny, 2015; https://davidakenny.shinyapps.io/APIMoM/), was used to perform the APIM moderation effects (i.e., the potential moderation effects of relationship satisfaction).

## Results

The MANOVA revealed no statistically significant impact of the sociodemographic and illness-related variables (i.e., type and stage of cancer, type of treatment) on the dependent variables, Wilks λs < 0.95, *F*s (6, 40) < 1.30, *p* > 0.05, η^2^s < 0.15. Patient age and relationship duration were not significantly related to DC, relationship satisfaction, or illness representations (*r* < 0.20, *p* > 0.05). With respect to partner age, only one statistically significant correlation was found with own treatment control (*r* = 0.30, *p* < 0.01). Also, patient and partner age were not related to (patient or partner) relationship satisfaction (*r* < 0.10, *p* > 0.05).

**Figure 1:**
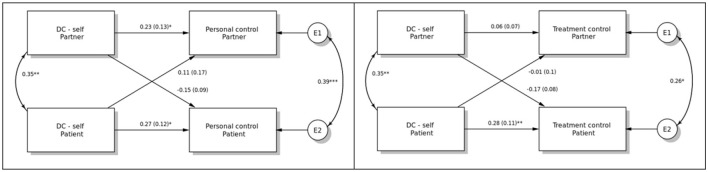
The dyadic effects of dyadic coping/support provided by self to the other member of the couple (DC-self) on personal and treatment control. *p < 0.05, **p < 0.01, ***p < 0.001.

Regarding the APIM results, the dyadic effects of the support provided by self to the other member of the couple (DC-self) on illness representations are illustrated in Figure 1. Only actor effects of patient and partner DC-self on own personal control were found to be significant (b = 0.27 and 0.23, *p* < 0.05, respectively). Also, only a patient-actor effect was found with respect to own treatment control (b = 0.28, *p* < 0.01). No significant effects were found for the timeline.

**Figure 2:**
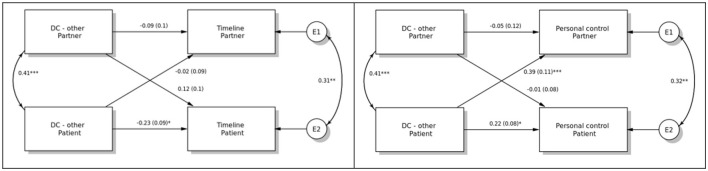
The dyadic effects of dyadic coping/support provided by the other member of the couple (DC-other) on timeline and personal control. *p < 0.05, ** *p* < 0.01, ***p < 0.001.

The dyadic effects of the perceived support provided by the other member of the couple on illness representations are presented in Figure 2. Patient perception of support provided by their partner was related to their own representation of illness timeline (b = −0.23, *p* < 0.05) and positively to their own and also their partners' representation of personal control over illness (b = 0.22, *p* < 0.05, and 0.39, *p* < 0.001, respectively). No other effects were detected.

**Figure 3:**
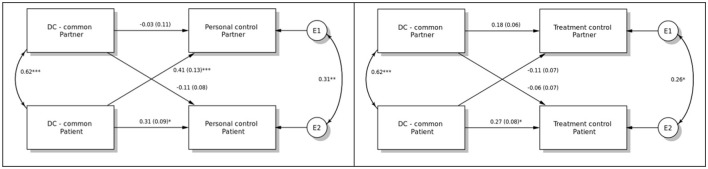
The dyadic effects of common dyadic coping (DC-common) on personal and treatment control. *p < 0.05, **p < 0.01, ***p < 0.001.

In Figure 3, the dyadic effects of common DC on illness representations are illustrated. Patient common DC was related to own representation of personal control (actor effect; b = 0.31, *p* < 0.05), as well as to partner corresponding representation (partner effect; b = 0.41, *p* < 0.001). It was also related to its own representation of treatment control (actor effect; b = 0.27, *p* < 0.05). There were no other statistically significant effects.

Finally, there is no evidence that the relationships between DC and illness representations were moderated by patient or partner relationship satisfaction, as no statistically significant effects were revealed (*B*_s_ < 0.22, *p* > 0.05, 95% confidence intervals = −0.47 to 0.49). Moreover, the comparison between the models that included possible interaction effects and those without interaction effects showed no statistically significant differences ($\chi_{\text{s}}^{2}$(4) < 6.90, *p* > 0.05; RMSEA across comparisons >0.09).

## Discussion

The present study examined the relationship between DC and illness representations in recently diagnosed female cancer patients and their partners from a dyadic regulation perspective. The primary objective of the study was to investigate the impact of DC on perceptions of personal control, treatment control, and timeline related to the illness. This included examining how the DC strategies adopted by one member of the dyad were related to the illness representations of both themselves and the other member of the dyad. The study aimed to explore the extent to which DC influenced beliefs about personal control (the belief that one's actions can help cure the illness, i.e., “in what extend do I believe that things I can do will help curing my illness”), treatment control (the perception that the treatment will lead to a cure, i.e., “in what extend do I perceive that my treatment will cure my illness”), and the timeline of the illness (the perception of its duration, i.e., “how I perceive the duration of my illness”). The focus was on understanding how the perceptions of DC within the couple were related to these representations in both patients and partners in a dyadic regulatory manner.

Additionally, it aimed to examine the possible moderating role of relationship satisfaction in the relationship between DC and representations of illness for both the patient and partner.

The findings provided partial support with regards to the hypothesis that a sense of more support received by the other member of the couple will be related to a more positive representation of illness for both parties. The patient evaluations of DC were related to more positive own illness representations, but this was not the case for partners. Partners' evaluations of DC were not related to either their own or patients' representations of illness.

At the same time and to the extent that the (dyadic) support provided by self to the other member of the couple also reflects the broader sense of dyadic support available in the couple, our hypothesis that its relation to the illness representations will be related to more positive representations was confirmed in some cases. Patients' evaluations of DC were related to their partners higher levels of control over the illness. Moreover, patients' representations of higher levels of personal control—thus having a sense of being able to control their illness—were related to partners' sense of receiving support from the other/patient. As far as partners are concerned, their evaluations of DC were not related to patients' representations of illness or to their own representations.

The present findings are in accordance with previous findings, which show that individuals develop their understanding of illness based on other resources, such as all their previous experiences in life, information they have received up to the moment of the diagnosis, available coping resources. In other words, illness representations are formatted in the whole context of all the personal experiences of the individual and not only through the experience of the illness *per se* (Leventhal et al., 1997, 2016). To better comprehend the dyad and its dynamics, we need to take into account the disease, the family, medical, and social contexts while specifying the relations between the patient and his/her life partner, each entity with its own characteristics in terms of history, transactional variables, and criteria (Hasdenteufel and Quintard, 2022). Furthermore, the authors suggest that there are three concepts fundamental to understanding the processes of the dyad that fit perfectly into the conception presented above, “communication,” “reciprocal influence,” and “patient-caregiver congruence” (Hasdenteufel and Quintard, 2022). Elements of “reciprocal influence” are examined in the present study between two members of the “patient” and “caregiver” dyad, in the sense that one's perceptions of illness and coping are influenced by the other's. Additionally, partners may be too focused on the illness. So, even if the broader support coming from their patients is there, they cannot see its importance or take it for granted. It may be that they mainly focused on aspects of the illness in this initial phase of the illness trajectory, and therefore their evaluations of coping were not related to patients' representations of illness. On the other hand, patients' evaluations of coping were related to their own representations of illness, implying that patients are more focused on their current new experience, especially at this early stage of the diagnosis.

It could be argued that the present findings may also reflect differences in the behaviors or attitudes involved in different roles (i.e., patient vs. partner), as explained above, or specific gender issues. In our study, all patients were women and all partners were men, and no partner or actor effects between DC and representations were found for the partners/men. This could be explained by specific gender role stereotypes, where partners' coping resources may be influenced by other factors outside of the spousal dyad as well. Greece has undergone major changes in the past 35 years. While previously Greek culture was described as more traditional with stronger gender roles (Hofstede, 1980), it seems that more recently Greeks score lower on gender empowerment measures and have more egalitarian attitudes. This is especially evident in studies that focus on participation in household and childcare (Maridaki-Kassotaki, 2000; Apparala et al., 2003). In certain cultural contexts, such as Greek culture, the involvement of extended family members may lead to a weaker bond between the spouses and potential conflicts (Georgas et al., 2001). Consequently, the strength of the couple's relationship may be different than expected, and other family members could influence the couple's adjustment. Additionally, a meta-analysis conducted by Hagedoorn et al. (2008) found that in studies exclusively involving female patients, the women reported higher distress levels compared to their partners. Conversely, in studies exclusively involving male patients, the partners reported higher distress levels than the patients themselves. These findings suggest that gender differences should be taken into account, particularly when studying patients of a specific gender. Although our study did not reveal significant differences between females and males, it is possible that their roles in the illness trajectory have subtle differences that require further exploration. Thus, future research should focus on gathering additional information about the factors that may affect gender roles as well as factors referring to the different illness-related roles, such as those of the “patient” and “caregiver.”

Although in partners there was no relationship found between DC and representations of illness, higher levels of DC in the patients were related to higher levels of control for the partners. An explanation is that partners may feel that the patients who report DC have the sense that they somehow can control their illness by providing support, and this may be affecting their own perceptions of control too. Nevertheless, this can be a rather tentative explanation and warrants further research.

Contrary to the fact that the dimension of control was related to DC, the timeline was not found to be related to DC for either patients or partners. This may be explained by the fact that the dimension of the timeline may include more “solid” characteristics, like illness facts and response to treatment. Whereas, the dimension of control may include more “individual” characteristics that can dynamically be inter-related with other factors and be open to change, such as attitudes, for example. Timeline and the way it is assessed in the particular questionnaire (IPQ-R) may also be vague concepts, especially in the case of cancer patients. For example, the item “My illness will last a short time” may be interpreted in different ways by cancer patients. As Benyamini and Karademas (2019) suggested, the development of this instrument may have led to specified definitions of representations, and maybe this is the case for the dimension of timeline. Although the authors of the questionnaire (Moss-Morris et al., 2002) have suggested that the items can be adjusted according to the different illness populations of each study, this is not always the case, and we did not apply it either. So, maybe future studies can assess the timeline using more accurate phrases related to cancer.

The second hypothesis was not supported by the findings. Unexpectedly, the relationship satisfaction did not moderate the relationships between DC and representations of illness for either patients or partners. This may be due to the following: it is possible that some aspects of relationship satisfaction have a greater impact on representation than others. For example, satisfaction from the communication of emotions in a relationship may be a stronger predictor than satisfaction from engaging in more common coping behaviors, which, at the same time, are both considered good quality characteristics in a relationship. DC may be one of the composing factors of relationship satisfaction and may be incorporated into the concept of satisfaction. The idea that “dyadic relationship wellbeing could be covered under relationship satisfaction (RS)” has also been suggested by Chen et al. (2021). Their findings suggest that the relationship satisfaction can encompass various aspects of the dyadic relationship's overall wellbeing. Therefore, the moderation may not be obvious when assessed as a separate concept. Communication of emotions or taking responsibility for partners' tasks may enhance relationship satisfaction and may need further focus, as Stefanult et al. (2021) have suggested. Another explanation for the lack of moderation effects of relationship satisfaction between DC and representations might be the modest sample size of the study, which could not allow the detection of medium- or small-sized effects.

There are limitations in our study concerning (a) the number of couples that participated was rather modest; (b) the fact that the data collection took place during the COVID-19 pandemic—when access to the hospital was restricted—may have impacted the recruitment process and the results; and (c) this is a cross-sectional study, and as such, it cannot provide any evidence about the direction of the relationships examined. A longitudinal approach is needed to provide us with more information on the process and the ways that DC may determine illness representations. Finally, our study focused only on certain illness representations and certain aspects of DC (e.g., forms of negative DC were not assessed).

Still, the findings of this study underline the importance of adopting a dyadic regulation perspective in relevant studies and also bear some considerable clinical implications.

By recognizing the link between DC and illness representations of control and the illness timeline, healthcare professionals can focus on interventions that promote effective DC strategies. Encouraging partners to engage in supportive behaviors, active involvement in treatment-related decisions, and joint problem-solving can help foster a sense of control. This understanding can guide the development of couple-based interventions aimed at enhancing coping and improving psychosocial outcomes for both patients and partners in the context of cancer or other chronic illnesses.

Overall, the present findings point out the significant relationship between DC and illness representations, and thus they underline the importance of DC in both the patient's and partner's experience of illness. It has been well documented that illness representations and the entire experience of illness do not take place in a vacuum but are rather linked to the patients' (and partners') lives (Leventhal et al., 1997, 2016). Still, the processes related to dyadic regulation are uncharted and sometimes difficult to grasp (i.e., our finding that partners' illness representations are not related to their own evaluations of DC or that relationship satisfaction does not moderate the relationship between DC and illness representations). In this respect, the present study sheds some more light on understanding the complexity of patients' and their partners' adaptation to cancer. According to the present findings, in clinical interventions with couples facing the cancer experience and focusing on their coping strategies, representations of illness should be introduced, discussed, and maybe reframed more functionally to improve psychological adjustment.

## Data availability statement

The original contributions presented in the study are included in the article/supplementary material, further inquiries can be directed to the corresponding author.

## Ethics statement

The study involving human participants was reviewed and approved by the Ethics Committee of the University of Crete, Greece and was conducted in accordance with the local legislation and institutional requirements. It was also approved by the Scientific Committee of the following hospitals: Elena Venizelou General & Maternity Hospital in Athens, Greece and Bank of Cyprus Oncology Centre, Nicosia, Cyprus. The patients/participants and their partners provided their written informed consent to participate in this study.

## Author contributions

ZG: data collection from Cyprus site, literature review, data interpretation, and writing original draft. CT: data analysis and data interpretation. EK and AP: conceptualization, investigation, methodology, and editing. AP: supervision and project administration. All authors contributed to manuscript revision and approved the submitted version.
